# Pneumococcal colonization and severity of pneumonia in hospitalized Cambodian children following introduction of the 13-valent pneumococcal conjugate vaccine

**DOI:** 10.1016/j.ijregi.2023.05.005

**Published:** 2023-05-21

**Authors:** Thyl Miliya, Chansovannara Soputhy, Phana Leab, Pisey Tan, Sena Sao, James D. Heffelfinger, Nyambat Batmunkh, Vichit Ork, Md. Shafiqul Hossain, Nicholas P.J. Day, Claudia Turner, Paul Turner

**Affiliations:** a Cambodia Oxford Medical Research Unit, Angkor Hospital for Children, Siem Reap, Cambodia; b Regional Office for the Western Pacific, World Health Organization, Manila, Philippines; c National Immunization Programme, Ministry of Health, Phnom Penh, Cambodia; d World Health Organization, Phnom Penh, Cambodia; e Mahidol Oxford Tropical Medicine Research Unit, Faculty of Tropical Medicine, Mahidol University, Bangkok, Thailand; f Centre for Tropical Medicine and Global Health, Nuffield Department of Medicine, University of Oxford, Oxford, UK

**Keywords:** *Streptococcus pneumoniae*, Paediatric, Colonization, Pneumonia, Vaccine

## Abstract

**Objectives:**

This study sought to characterize pneumococcal colonization and clinical/radiological features in Cambodian children admitted to hospital with an illness compatible with pneumonia following national introduction of the 13-valent pneumococcal conjugate vaccine (PCV13).

**Methods:**

Children aged 0–59 months admitted to Angkor Hospital for Children who met the World Health Organization (WHO) case definition for clinical pneumonia were enrolled over a 3-year period. Clinical, radiological and vaccination data were collected at enrolment. A nasopharyngeal swab was collected for detection of pneumococcal colonization using the WHO standard culture method.

**Results:**

Between 1 September 2015 and 31August 2018, 2209 analysable illness episodes were enrolled. Pneumococci were detected in 943/2209 (42.7%) children. PCV13 serotypes were detected less frequently in children who had been vaccinated appropriately for their age compared with undervaccinated children: 309/567 (53.6%) vs 216/342 (63.2%) (*P*=0.006). Age-appropriate PCV13 vaccination was negatively associated with hypoxic presentation [adjusted odds ratio (aOR) 0.72, 95% confidence interval (CI) 0.60–0.87; *P*=0.0006] and primary endpoint pneumonia on chest x ray (aOR 0.69, 95% CI 0.54–0.90; *P*=0.006).

**Conclusions:**

The introduction of PCV13 in Cambodia was associated with a decline in vaccine serotype nasopharyngeal colonization, and clinical and radiological severity in children hospitalized with clinical pneumonia.

## Highlights

• Introduction of the 13-valent pneumococcal conjugate vaccine (PCV13) led to declines in vaccine serotype pneumococcal colonization.
• PCV13 vaccination was negatively associated with hypoxic pneumonia.
• PCV13 vaccination was negatively associated with radiologic pneumonia.

## Introduction

Pneumonia is a leading cause of childhood mortality in low- and middle-income countries, with *Streptococcus pneumoniae* being the dominant bacterial pathogen [1,2]. Pneumonia was the presenting clinical syndrome for 81% of almost 300,000 pneumococcal deaths in children aged <5 years in 2015 [3]. Although the introduction of multi-valent pneumococcal conjugate vaccines (PCV7–PCV13) has reduced the overall burden of infection, non-vaccine serotype disease persists and may erode this reduction in disease burden [4].

Defining the impacts of PCVs on the burden and aetiology of pneumonia is difficult as pneumonia remains a diagnostic challenge in children, and microbiological confirmation cannot be obtained in most cases [1,5]. In contrast, it is relatively straightforward to detect pneumococcal nasopharyngeal colonization and, with careful study design, it is possible to identify the serotypes that are more commonly detected in children with pneumonia compared with healthy controls [6,7]. Using such data from Israel, it was estimated that two primary doses of PCV13 plus a booster provided >80% protection to children aged 12–35 months against chest-x-ray-confirmed pneumonia attributable to vaccine serotypes [8].

PCV13 was added to the Cambodian national immunization schedule in January 2015, with a 3+0 dosing schedule (doses at 6, 10, 14 weeks and no booster) and no catch-up campaign. The invasive pneumococcal disease and colonization landscape before the introduction of PCVs [7,[9], [10], [11]], and the impact of vaccine introduction [12], have been described previously. However, there are no data describing the impact of vaccine introduction on pneumococcal colonization in children with pneumonia in Cambodia.

The main aim of this study was to characterize pneumococcal colonization in Cambodian children aged <5 years admitted to a paediatric referral hospital with a clinical syndrome compatible with pneumonia in the first 3 years following national introduction of PCV13. An additional aim was to compare colonization characteristics with previously published data from outpatient children swabbed during cross-sectional surveys over the same time period [12]. Finally, this study sought to identify early signals of the impact of PCV13 on clinical and radiological pneumonia in this population.

## Methods

### Study site

Angkor Hospital for Children (AHC) is a non-governmental paediatric referral hospital providing free health care to children aged <16 years from across Cambodia. The main hospital site is located in Siem Reap city and has approximately 80 beds, with around 125,000 outpatient visits and 3300 inpatient admissions annually. Cambodia has a tropical climate, with monsoon rains typically occurring between May and October each year (‘rainy season’).

### Study participants

Children aged 0–59 months admitted to AHC with an acute illness meeting the World Health Organization (WHO) case definition for clinical pneumonia [13] (Table 1), and not enrolled into the study within the preceding 14 days, were eligible for recruitment. At enrolment, details of the presenting illness, past medical history, vaccination status (by review of handheld record or from parental recall) and recent antibiotic exposure (defined as the week before hospital admission) were recorded and a nasopharyngeal swab (NPS) was collected. At hospital discharge, details of inpatient treatment were recorded along with clinical outcome. If a chest x ray was ordered by the treating paediatrician, the digitized film was reviewed by one of the study team (CT or PT, blinded to clinical data) and interpreted following the guidance of the WHO Radiology Working Group [14].

**Table 1:** Case definitions of clinical pneumonia used in the study [13].

**Pneumonia**
Cough and/or difficulty breathing
*AND*
Tachypnoea when calm (fast breathing): • ≥60 breaths/min if aged <2 months • ≥50 breaths/min if aged 2–11 months • ≥40 breaths/min if aged 12–59 months
**Severe pneumonia**
Cough and/or difficulty breathing
*AND*
At least one of: • Respiratory distress • Chest indrawing (supracostal, subcostal, substernal or intercostal recession) • Stridor when calm (noisy inspiration) • General danger sign • Inability to breast feed or drink • Vomiting everything • Convulsions • Prostration/lethargy (abnormally sleepy or difficult to wake)

### Detection of pneumococcal colonization

Flocked nylon tipped NPSs (Medical Wire and Equipment, Corsham, UK) were processed according to the updated WHO detection protocol [12,15]. Swab tips were excised into 1-mL skimmed milk-tryptone-glucose-glycerol medium (STGG; prepared in-house), stored immediately in a cool box, and frozen at -80°C within 8 h of collection. After thawing, 100 µL of NPS-STGG was cultured overnight at 36°C in 5% CO_2_ on sheep blood agar supplemented with 5 mg/L gentamycin (Columbia agar base, Oxoid, Basingstoke, UK; prepared in-house). Alpha-haemolytic colonies were confirmed as *S. pneumoniae* by optochin susceptibility, with bile solubility used to confirm intermediate results. Pneumococcal isolates were serotyped by latex agglutination, with Quellung confirmation of equivocal results [16]. If serotype could not be confirmed in a phenotypically typical pneumococcal isolate, by either latex agglutination or Quellung typing, the serotype was recorded as ‘unresolved’. Antimicrobial susceptibility testing (AST) was performed using disk diffusion (chloramphenicol, clindamycin, erythromycin, co-trimoxazole, tetracycline; Oxoid) and Etest minimum inhibitory concentration (MIC; penicillin, ceftriaxone; bioMérieux, Marcy-l'Etoile, France), as described previously [12]. AST results were interpreted using the 2018 breakpoint guidelines of the Clinical Laboratory and Standards Institute [17,18]. Resistance to penicillin and ceftriaxone was defined as MIC ≥0.12 µg/mL and ≥1 µg/mL, respectively. For the other agents, ‘intermediate’ resistance was reclassified as ‘susceptible’ to give a conservative estimate of resistance where MIC values were not measured. Multi-drug resistance (MDR) was defined as resistance to three or more agents, with clindamycin/erythromycin and benzylpenicillin/ceftriaxone counting as single agents.

### Data analysis

Children were classified as PCV13 vaccinated if they were aged 0–11 months and had received at least two doses of PCV13, or if aged ≥12 months and had received at least one dose of PCV13 [19]. Hypoxia was defined as oxygen saturation <90% in room air at initial assessment or if supplemental oxygen or ventilatory support was administered during hospitalization. Serotypes 1, 3, 4, 5, 6A, 6B, 7F, 9V, 14, 18C, 19F, 19A and 23F were classified as PCV13 vaccine serotypes, with all other serotypeable pneumococci classified as non-vaccine serotypes, and phenotypically unencapsulated isolates were classified as non-typeable. Given the nature of their capsules, serotypes 15B and 15C were combined for analysis and denoted as 15B/C.

Categorical variables were compared using Chi-squared test, and trends were assessed using Cochran-Armitage test. Continuous variables were summarized by median and interquartile range (IQR).

In order to assess the relationship between PCV13 vaccination status and clinical or radiological severity at presentation, two multi-variable logistic regression models were fitted, with hypoxia or presence of primary endpoint pneumonia on chest x ray as the dependent variable. For both models, independent predictors defined a priori were PCV13 vaccination status, age (in months), sex, presence of comorbidities [human immunodeficiency virus infection, tuberculosis (currently/previously treated), asthma, heart disease and splenectomy], recent antimicrobial exposure, household size, and season of presentation. Vaccine efficacy against hypoxia or primary endpoint pneumonia was calculated as: [1–odds ratio (OR) x 100] [19].

To determine differences in pneumococcal serotype colonization between children hospitalized with clinical pneumonia compared with children with minor illnesses (as a proxy for healthy children), data from the pneumonia cohort were compared with data from four cross-sectional colonization surveys conducted at AHC outpatient department between August 2015 and January 2018 [12]. Swab, culture and pneumococcal characterization protocols used in these surveys (subsequently described as the ‘carriage cohort’) were identical to those described here. Carriage of pneumococcal serotypes in the pneumonia cohort was compared with the non-pneumonia carriage cohort by logistic regression, controlling for PCV13 immunization status, recent antibiotic exposure and age group (<2 months, 2–11 months, 12–59 months). ORs were calculated relative to carriage of serotype 6B, which was common to both cohorts [6].

Analyses were performed using R Version 4.2.2 [20], with the following packages: DescTools Version 0.99.47, ggbreak Version 0.1.1, gtsummary Version 1.6.3, lubridate Version 1.9.0, readxl Version 1.4.1, summarytools Version 1.0.1 and tidyverse Version 1.3.2.

## Results

### Study participants

Between 1 September 2015 and 31 August 2018, 7288 inpatient admissions were screened for study eligibility. Of those, 2907 met the enrolment criteria and 2265 were enrolled. After exclusion of 56 episodes [WHO pneumonia case definition not met (*n*=11), not an acute illness (duration of illness >14 days, *n*=42), or missing NPS (*n*=3)], there were 2209 analysable illness episodes (Fig. S1, see online supplementary material).

Enrolment varied considerably by season, with peaks seen towards the end of the rainy season each year (Fig. S2, see online supplementary material).

The median age at presentation was 9 months (IQR 3–15), and 41.8% (922/2209) were female. PCV13 vaccination status was known in 2106/2209 (95.3%) cases: 1168 (55.5%) had been vaccinated appropriately for their age. The proportion of children who had been vaccinated appropriately for their age increased each year: 24.6% (75/305) in 2015, 52.1% (340/652) in 2016, 64.5% (485/752) in 2017, and 67.5% (268/397) in 2018 (*P*<0.0001). Demographic and clinical details are summarized in Table 2.

**Table 2:** Characteristics of 2209 enrolled and analysable children.

Characteristic	*n*=2209^a^
Sex	
Female	922 (42%)
Male	1287 (58%)
Age (months)	9 (3–15)
Age category	
<2 months	397 (18%)
2–11 months	1016 (46%)
12–59 months	796 (36%)
PCV13 vaccination status	
Under-vaccinated^b^	938 (45%)
Vaccinated	1168 (55%)
Unknown	103
Comorbidities^c^	
No	1879 (85%)
Yes	330 (15%)
Household size	5 (4–7)
Unknown	1
Duration of illness (days)	4 (3–6)
Unknown	3
Admitted from	
Healthcare facility	283 (13%)
Home	1926 (87%)
WHO clinical pneumonia category	
Pneumonia	267 (12%)
Severe pneumonia	1942 (88%)
Clinical discharge diagnosis of pneumonia or bronchiolitis	
No	572 (26%)
Yes	1637 (74%)
WHO chest x-ray interpretation	
No consolidation/infiltrate/effusion	1193 (54%)
Other infiltrate/abnormality	498 (23%)
Primary endpoint pneumonia	298 (13%)
Uninterpretable	26 (1.2%)
No chest x ray	194 (8.8%)
Respiratory support (maximum level required)	
None	1184 (54%)
Oxygen	408 (18%)
CPAP	361 (16%)
Mechanical ventilation	256 (12%)
Hospitalization outcome	
Discharged alive	2163 (98%)
Discharged dead or moribund	46 (2.1%)

WHO, World Health Organization; CPAP, continuous positive airway pressure.

a *n* (%) or median (interquartile range).

b Includes children partially and non-vaccinated.

c Comorbidities assessed: human immunodeficiency virus infection, tuberculosis (currently/previously treated), asthma, heart disease and splenectomy.

### Severity of pneumonia

Most cases (1942/2209, 87.9%) met the WHO criteria for severe pneumonia, and almost half (1025/2209, 46.4%) required respiratory support. The presence of hypoxia was more common in undervaccinated children compared with those who had been vaccinated appropriately for their age [478/938 (51.0%) vs 489/1168 (41.9%); *P*<0.0001]. There was no temporal trend in the presence of hypoxia, which varied between 39.6% (264/667) in 2016 and 52.4% (161/307) in 2015 (*P*=0.4). After adjustment for age, sex, comorbidities, recent antimicrobial exposure, household size and season of presentation, age-appropriate PCV13 vaccination remained negatively associated with hypoxic presentation [adjusted OR 0.72, 95% confidence interval (CI) 0.60–0.87; *P*=0.0006; Table S1, see online supplementary material]. Vaccine effectiveness against hypoxic pneumonia was 28% (95% CI 13–40%).

Almost all cases had an interpretable chest x ray (1989/2209, 90.0%; 194 did not have a chest x ray and 26 had an uninterpretable film): 15.0% (298/1989) were categorized as primary endpoint pneumonia and 25.0% (498/1,989) were categorized as other infiltrate. Primary endpoint pneumonia was detected more frequently in undervaccinated children compared with those who had been vaccinated appropriately for their age [146/849 (17.2%) vs 134/1052 (12.7%); *P*=0.008]. The presence of primary endpoint pneumonia on chest x ray declined over time: 18.9% (58/307) in 2015, 13.6% (91/667) in 2016, 12.5% (101/811) in 2017, and 10.6% (45/424) in 2018 (*P*=0.007). After adjustment, age-appropriate PCV13 vaccination remained negatively associated with primary endpoint pneumonia on chest x ray (adjusted OR 0.69, 95% CI 0.54–0.90; *P*=0.006; Table S2, see online supplementary material). Vaccine effectiveness against primary endpoint pneumonia was 31% (95% CI 10–46%).

### Pneumococcal colonization

**Fig. 1:**
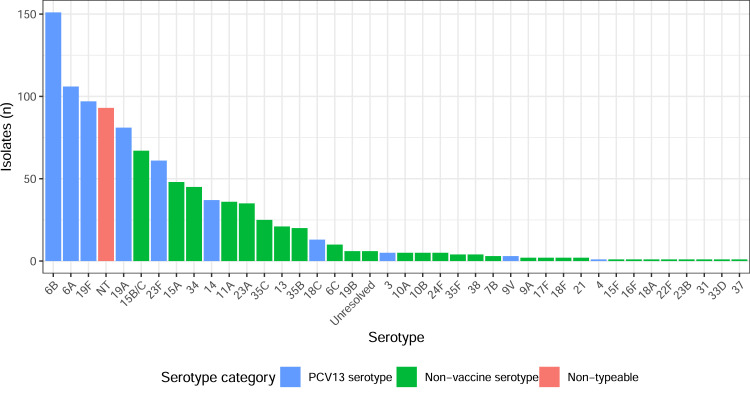
Pneumococcal serotypes identified from 943 colonized children, by vaccine serotype category. Unresolved, phenotypically encapsulated colonies where serotype could not be determined by latex agglutination and Quellung. PCV13, 13-valent pneumococcal conjugate vaccine.

Overall, 943/2209 (42.7%) of children were colonized by at least one pneumococcal serotype: more than one serotype was detected in 66 children, yielding a total of 1009 pneumococcal isolates for further study. Just over half of those colonized (540/943, 57.3%) were colonized by a PCV13 serotype, 356/943 (37.8%) were colonized by a non-vaccine serotype, and 92/943 (9.8%) were colonized by a non-typeable pneumococcus (Figs. 1 and S3, see online supplementary material).

Confirmed antibiotic use in the week before hospitalization had a small impact: pneumococci were detected in 849/1957 (43.4%) children with no recent antibiotic use and in 94/249 (37.8%) of children who had received an antibiotic in the preceding week (*P*=0.104). Administration of an antibiotic at hospital admission had a greater impact on pneumococcal detection: pneumococci were detected in 760/1450 (52.4%) children where the NPS was taken pre-antibiotic and in 183/759 (24.1%) children where the NPS was taken post-antibiotic (*P*<0.0001). The 10 most frequently carried serotypes were identical in the entire cohort and in the subset who had no recent or pre-swab antibiotic exposure, although the serotype rank order varied. Comparing antibiotic-exposed children with non-exposed children revealed differences in the dominant serotypes: eight of the top 10 serotypes were identical, with serotypes 14 and 34 only appearing in the non-exposed group, and serotypes 11A and 35C only appearing in the antibiotic-exposed group (Figs. S4 and S5, see online supplementary material).

PCV13 serotypes were detected less frequently in children who had been vaccinated appropriately for their age compared with undervaccinated children: PCV13 serotypes were detected in 216/342 (63.2%) undervaccinated children compared with 309/567 (53.6%) children who had been vaccinated appropriately for their age (*P*=0.006).

**Fig. 2:**
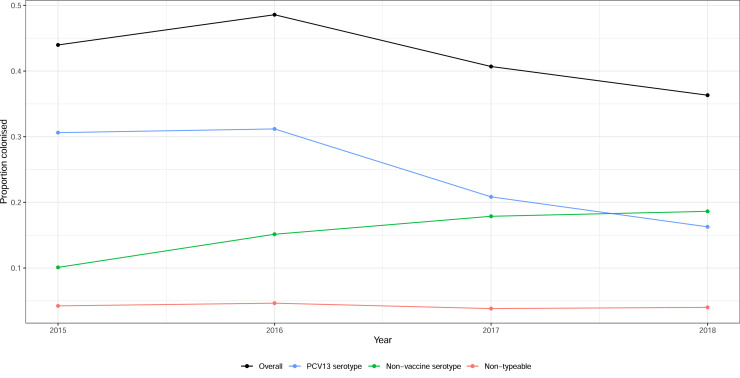
Pneumococcal colonization in 2209 cases of clinical pneumonia by year of enrolment. PCV13, 13-valent pneumococcal conjugate vaccine.

There was a decline in overall colonization rate over time, from 135/307 (44.0%) in 2015 to 154/424 36.3% in 2018 (*P*=0.0007). PCV13 serotype colonization declined [94/307 (30.6%) in 2015 to 69/424 (16.3%) in 2018; *P*<0.0001], whilst non-vaccine serotype colonization increased [31/307 (10.1%) in 2015 to 49/424 (18.6%) in 2018; *P*=0.001], and non-typeable pneumococcal carriage remained stable [13/307 (4.2%) in 2015 to 17/424 (4.0%) in 2018; *P*=0.6] (Figs. 2 and S6, see online supplementary material).

Overall, 828/1009 (82.1%) pneumococcal isolates were penicillin non-susceptible and 756/1009 (74.9%) were MDR. PCV13 serotype isolates were more likely to be penicillin non-susceptible than non-vaccine serotype or non-typeable isolates [514/555 (92.6%) vs 314/454 (69.2%); *P*<0.0001]. This was also true for MDR isolates [511/555 (92.1%, PCV13 serotypes) vs 245/454 (54.0%, non-vaccine serotypes and non-typeable isolates); *P*<0.0001].

### Pneumococcal serotype colonization in children hospitalized with pneumonia compared with outpatient children with minor illnesses

Colonization data from 1800 outpatient children aged 0–59 months (median 16 months, IQR 9–31) presenting with minor illnesses were compared with the pneumonia cohort described above. In this carriage cohort [12], 832/1755 (47.4%) children had been vaccinated appropriately for their age and 65/1798 (3.6%) children had received an antibiotic in the week before swabbing. The prevalence of pneumococcal colonization was 63.2% (1138/1800). Amongst pneumococcal colonized children, 608/1138 (53.4%) carried a PCV13 serotype, 511 (44.9%) carried a non-vaccine serotype, and 74 (6.5%) carried a non-typeable isolate.

**Fig. 3:**
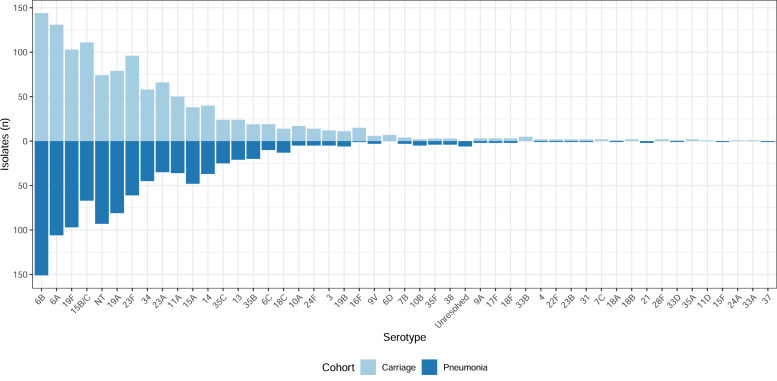
Colonizing pneumococcal serotypes by hospitalized pneumonia or outpatient carriage cohort.

The overall serotype distribution was similar between the pneumonia cohort and the carriage cohort (Fig. 3). A small number of serotypes were detected in a single cohort: 15F, 21 and 37 (pneumonia); and 6D, 7C, 11D, 18B, 24A, 28F, 33A, 33B and 35A (carriage). Controlling for age group, PCV13 vaccination status and recent antibiotic exposure, colonization by serotypes 10B, 14 or non-typeable pneumococci was significantly associated with clinical pneumonia, whereas colonization by serotypes 34, 15B/C, 23F, 13, 23A, 11A, 24F, 6C, 19B, 3, 10A, 16F, 6A, 19F and 24F was negatively associated with clinical pneumonia (Table S3 and Fig. S7, see online supplementary material).

## Discussion

This 3-year prospective study demonstrated the impacts of national introduction of PCV13 in Cambodia on pneumococcal colonization and clinical/radiological severity in hospitalized children meeting the WHO case definition for clinical pneumonia.

Overall, pneumococcal colonization was dominated by PCV13 serotypes, although there was a gradual decline in PCV13 serotype colonization from 2016, in parallel with the rising proportion of PCV13-vaccinated children. As shown by the previous outpatient cross-sectional surveys at the same surveillance site, this decline in PCV13 serotype colonization was mirrored by an increase in non-vaccine serotype colonization [12]. Whilst overall serotype distribution was similar between hospitalized children with pneumonia and outpatient children with minor illnesses, some differences were noted. Serotypes 10B, 14, 15F, 21 and 37 were more commonly detected in children with pneumonia, although the absolute numbers were small. Of these, only serotype 14 was documented as a cause of invasive pneumococcal infection over the same time period [12]. Interestingly, non-typeable pneumococci were also more commonly isolated in children with pneumonia, and this has been noted previously in Israel [6]. The explanation for this association is not immediately forthcoming, as non-typeable isolates are rarely associated with severe infections given the dominance of the polysaccharide capsule as a virulence factor [21].

Very few studies have been undertaken to document the changes in pneumococcal colonization in children with pneumonia following PCV introduction. Five years after introduction of PCV10 in Ethiopia, children who had received at least two doses of vaccine were less likely to be colonized by pneumococci (adjusted OR 0.37, 95% CI 0.15–0.92), and the dominant colonizers were the vaccine-related serotypes 6A and 19A plus non-vaccine serotype 16F [22]. In Nepal, colonization by vaccine serotypes decreased by 82% in children with clinical pneumonia aged <2 years in the 4 years following PCV10 introduction [23].

Antimicrobial resistance was common in colonizing pneumococcal isolates, and more so in PCV13 serotypes than non-vaccine serotypes. As has been seen in other settings, PCV13 introduction would thus be expected to result in a modest decrease in the burden of antimicrobial resistance associated with pneumococcal disease [24].

The data presented in the current study identify early impacts of PCV13 on hypoxic pneumonia and radiologically confirmed pneumonia, with both decreasing in frequency over the 3-year study period. Vaccine effectiveness was 28% (95% CI 13–40%) against hypoxic pneumonia and 31% (95% CI 10–46%) against primary endpoint pneumonia. Similar declines have been noted in other recent regional studies. In Laos, PCV13 was shown to have effectiveness of 23–37% against hypoxic pneumonia in hospitalized children aged <5 years [19]. Fiji introduced PCV10 in 2012 and, by time series analyses of 2007–2017 data from three public hospitals, identified reductions of 46% for hypoxic pneumonia and 25% for radiological pneumonia in children aged 2–23 months [25]. In contrast, in Nepal, the prevalence of primary endpoint pneumonia in children aged <5 years varied during the 4 years following PCV10 introduction: it was 40% lower than the pre-vaccine period after 3 years, but 1 year later had risen again and prevalence was comparable with the pre-vaccine period [23].

The major limitation of this study was that it was carried out in a single surveillance site over a relatively short time frame following vaccine introduction, so may not represent the situation across Cambodia. Robust pneumonia data for the pre-vaccine period were not available for comparison, meaning a time-series-based approach to determine changes in clinical or radiological severity was not possible. Although experienced readers used WHO Radiology Working Group guidelines to interpret chest x rays, the digitized films were not double read nor were results validated by a radiologist. However, the investigators who did read the films were clinicians with considerable experience reading radiographs in the context of clinical practice and pneumococcal studies. Pneumococcal serotype detection followed the updated standardized WHO guidelines and is thus comparable with other major studies [15]. However, culture-based detection of pneumococcal colonization may have less than optimal sensitivity, despite excellent specificity. Recent advances in molecular serotype detection by sequencing have demonstrated improved identification of serotypes associated with highly invasive diseases, notably serotype 1 which is rarely detected in colonization studies [26]. Finally, nasopharyngeal colonization data on pneumococcal serotypes were used as a proxy for pneumonia aetiology, rather than data from sputum cultures or more invasive samples, which necessitates caution regarding any inferences around pneumonia causality. Despite these limitations, clear signals of the impact of PCV13 on colonization and the severity of pneumonia were detected, and these were similar to findings from contemporaneous studies in other resource-limited locations.

## Conclusion

National introduction of PCV13 in Cambodia was associated with a decline in vaccine serotype nasopharyngeal colonization, and clinical and radiological severity in children hospitalized with clinical pneumonia. The increase in non-vaccine serotype colonization warrants ongoing surveillance to document the impact of this serotype replacement on disease characteristics.

## Funding

This work was supported by 10.13039/100004423WHO (Grant Nos TSA 201180619, TSA 201559881). This research was also funded in whole, or in part, by the 10.13039/100010269Wellcome Trust (Grant No. 220211). The funders had no role in the study design, data collection, data analysis, data interpretation or writing of the manuscript. For the purpose of open access, the author has applied a CC BY public copyright licence to any Author Accepted Manuscript version arising from this submission.

## Ethical approval

Written informed consent was obtained from the child's legally responsible representative prior to study enrolment. The study protocol was approved by AHC Institutional Review Board (0348/15), Cambodia National Ethics Committee for Health Research (210NECHR), WHO Western Pacific Regional Office Institutional Review Board (2015.6.CAM.1.EPI), and the University of Oxford Tropical Research Ethics Committee (559-15).

## Conflict of interest statement

None declared.

## Appendix Supplementary materials

Image, application 1
