# Supplementary material for: Pneumococcal colonization and severity of pneumonia in hospitalized Cambodian children following introduction of the 13-valent pneumococcal conjugate vaccine
Source: IJID Reg. 2023 May 21;8:9–15. doi: 10.1016/j.ijregi.2023.05.005 (PMC10423667; doi:10.1016/j.ijregi.2023.05.005)
Supplement: Supplementary file 1 [file mmc1.docx]

# Supplmentary material

# Pneumococcal colonisation and pneumonia severity in hospitalised Cambodian children following introduction of the 13-valent pneumococcal conjugate vaccine

Thyl Miliya, Chansovannara Soputhy, Phana Leab, Pisey Tan, Sena Sao, James D. Heffelfinger, Nyambat Batmunkh, Vichit Ork, Md. Shafiqul Hossain, Nicholas PJ Day, Claudia Turner, Paul Turner

**
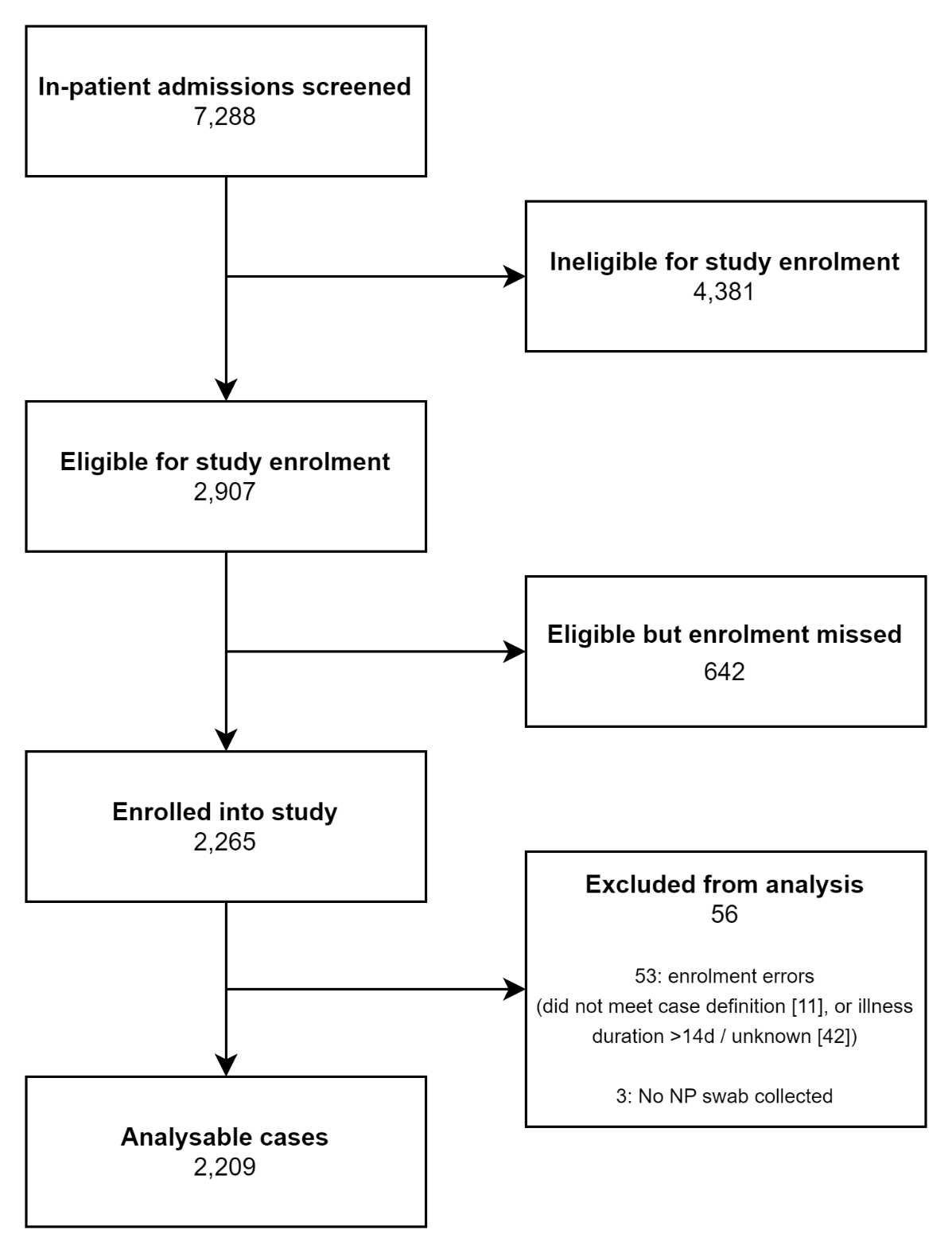
**

**Supplementary Figure 1. Study enrolment flowchart**


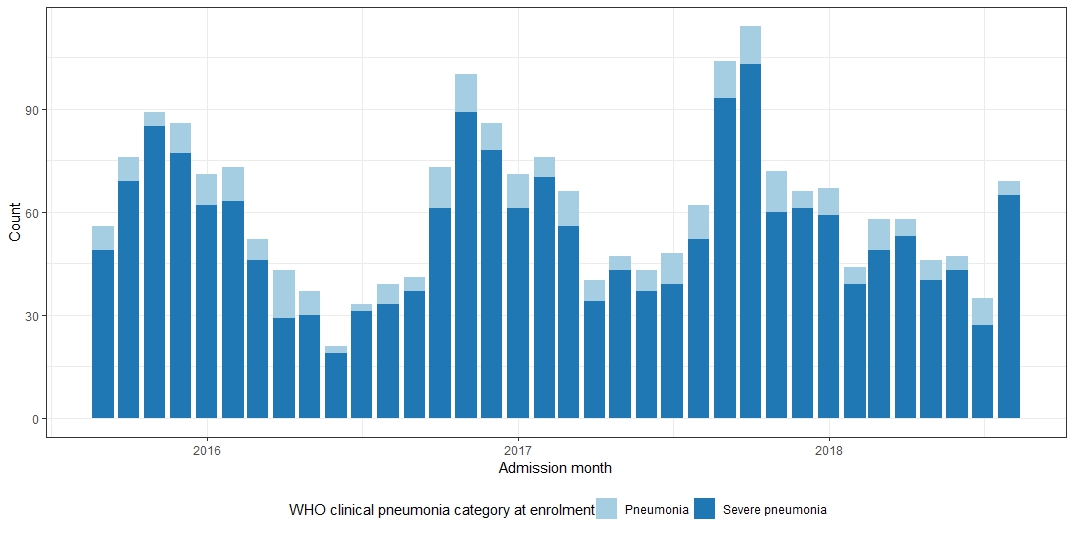


**Supplementary Figure 2. Study participant enrolment by month and WHO clinical pneumonia severity (n = 2,209)**

**
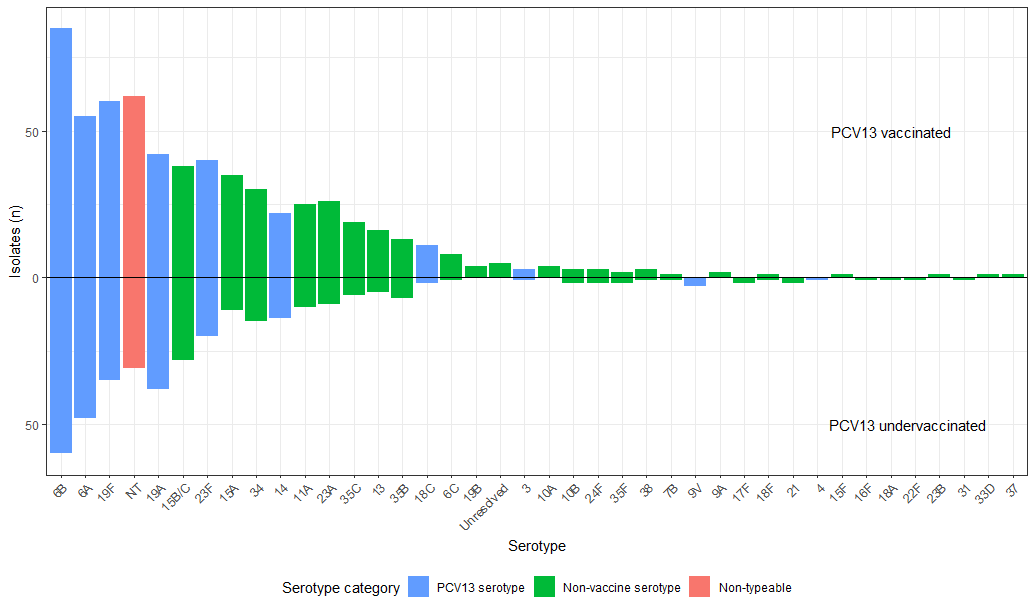
**

**Supplementary Figure 3. Pneumococcal serotypes identified from 918 colonised children stratified by PCV13 vaccination status, coloured by vaccine serotype category**

Vaccine status was known in 918/943 pneumococcus colonised children.


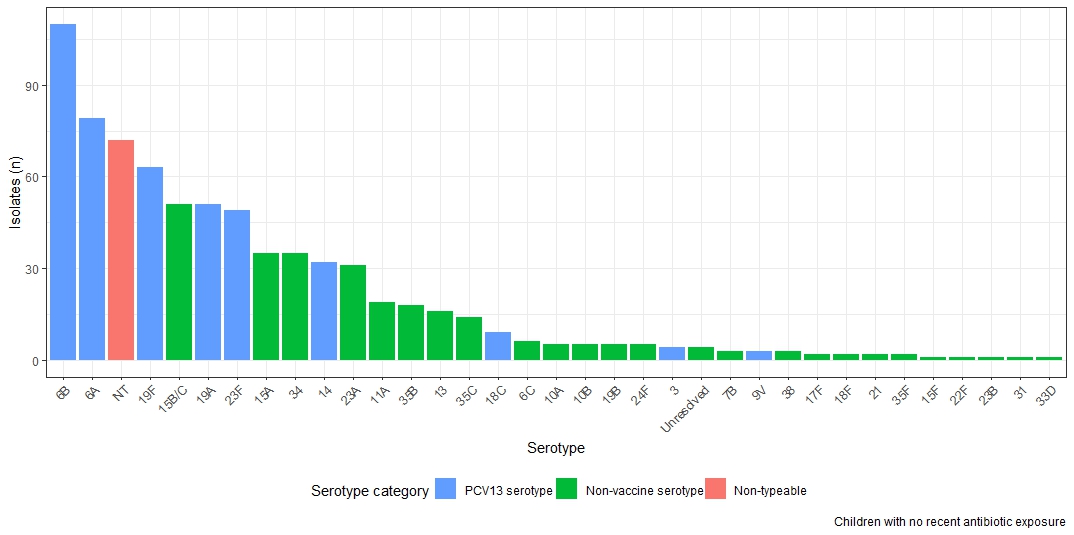


**Supplementary Figure 4. Pneumococcal serotypes identified from 691 colonised children with no antibiotic exposure in the week preceding admission (and nasopharyngeal swab collected prior to any admission antibiotics), coloured by vaccine serotype category**

Unresolved: phenotypically encapsulated colonies where serotype could not be determined by latex agglutination and Quellung.


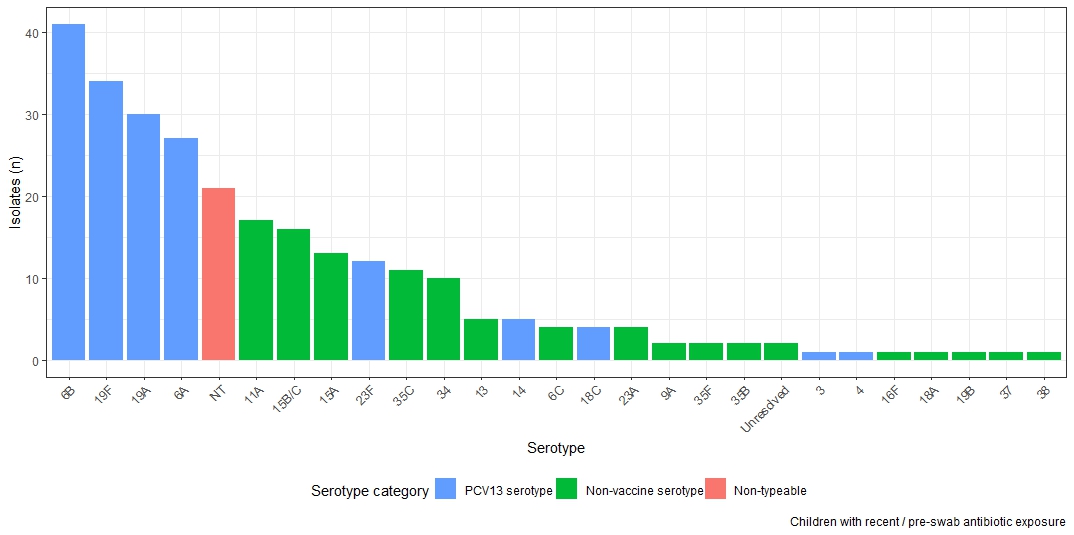


**Supplementary Figure 5. Pneumococcal serotypes identified from 252 colonised children with antibiotic exposure in the week preceding admission and / or nasopharyngeal swab collected after any admission antibiotics, coloured by vaccine serotype category**

Unresolved: phenotypically encapsulated colonies where serotype could not be determined by latex agglutination and Quellung.


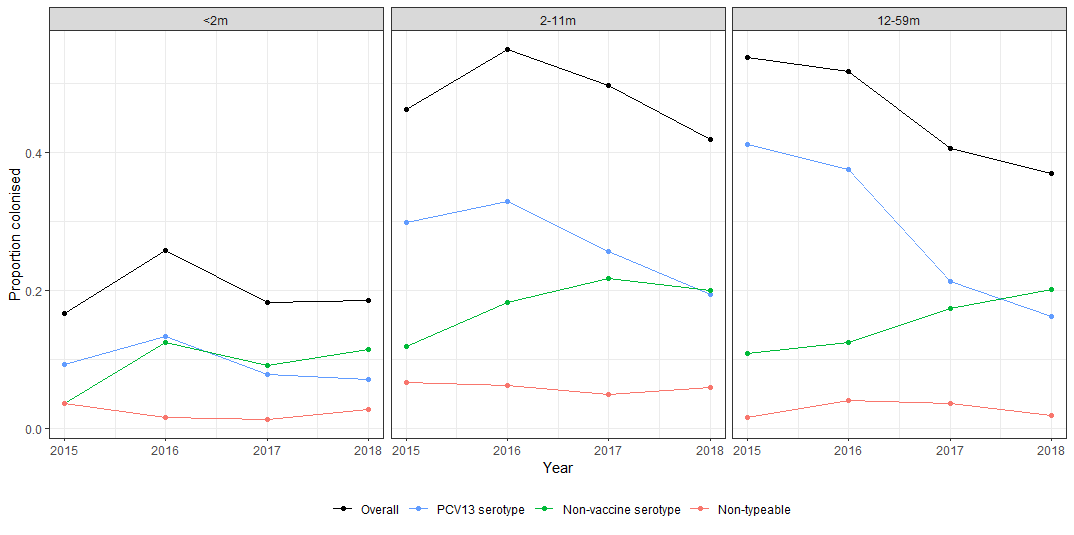


**Supplementary Figure 6. Pneumococcal colonisation in 2,209 clinical pneumonia cases by year of enrolment and WHO age group**


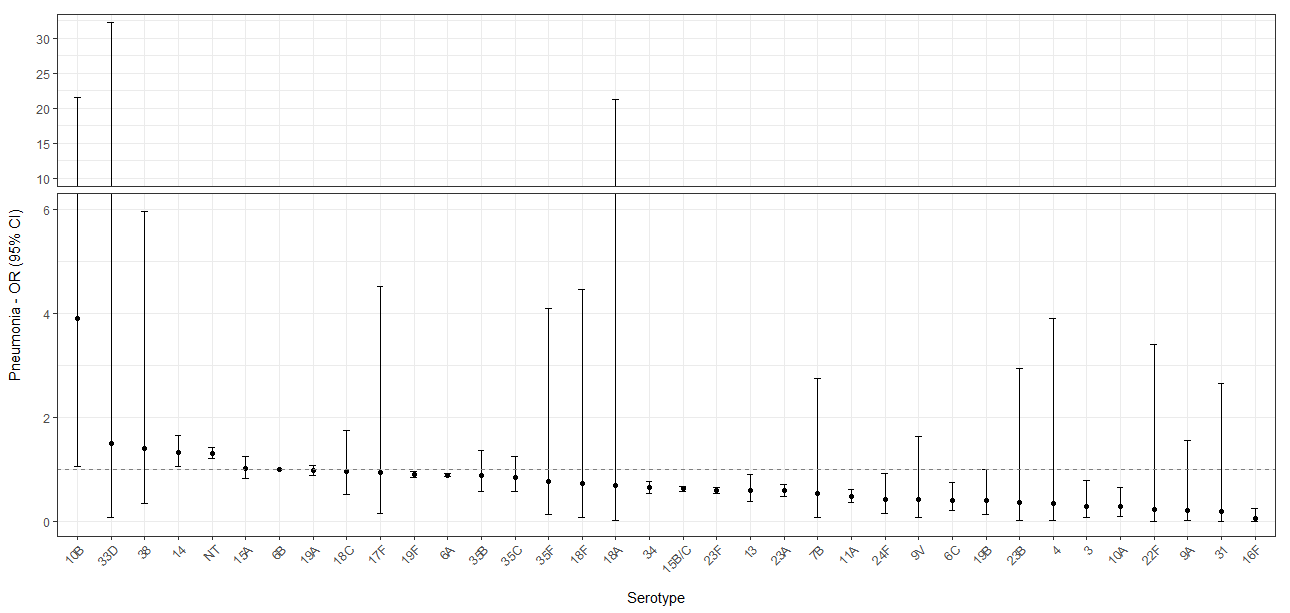


**Supplementary Figure 7. Pneumococcal serotype detection from nasopharyngeal swab, by cohort (pneumonia = 2,209 swabs; carriage = 1,800 swabs)**

Odds Ratios (OR) and associated 95% confidence intervals (CI) were adjusted for PCV13 immunisation status, recent antibiotic exposure, and age group and are presented relative to serotype 6B. An OR >1 indicates that detection of the serotype was more common in children presenting with clinical pneumonia.

| **Variable** | **Odds Ratio** | **95% Confidence Interval** | **p-value** |
| --- | --- | --- | --- |
| PCV13 vaccinated* | 0.72 | 0.60 – 0.87 | 0.0006 |
| Age (months) | 0.94 | 0.93 – 0.95 | <0.0001 |
| Sex (male) | 0.88 | 0.73 – 1.06 | 0.1774 |
| Comorbidity (any)† | 1.57 | 1.22 – 2.04 | 0.0006 |
| Antibiotic use in the week before presentation | 2.00 | 1.49 – 2.69 | <0.0001 |
| Household size (total number) | 1.00 | 0.97 – 1.04 | 0.8640 |
| Presentation in the rainy season (May – October) | 1.01 | 0.84 – 1.21 | 0.9192 |

**Supplementary Table 1. Results of a multivariable logistic regression model with hypoxia as the dependent variable**

The model includes data from 2,103 clinical pneumonia episodes. Hypoxia was defined as an oxygen saturation (SaO2) or <90% in room air at initial assessment or if supplemental oxygen / ventilatory support was administered during hospitalisation.

*PCV13 vaccinated defined as at least two doses of PCV13 (children aged between 0 – 11 months) or at least one dose of PCV13 (children aged ≥12 months).

†Comorbidities assessed: HIV infection, tuberculosis (currently / previously treated), asthma, heart disease, splenectomy.

| **Variable** | **Odds Ratio** | **95% Confidence Interval** | **p-value** |
| --- | --- | --- | --- |
| PCV13 vaccinated* | 0.69 | 0.54 – 0.90 | 0.0056 |
| Age (months) | 0.99 | 0.98 – 1.00 | 0.1232 |
| Sex (male) | 0.90 | 0.70 – 1.17 | 0.4201 |
| Comorbidity (any)† | 1.60 | 1.14 – 2.21 | 0.0055 |
| Antibiotic use in the week before presentation | 1.35 | 0.91 – 1.95 | 0.1253 |
| Household size (total number) | 0.94 | 0.89 – 0.99 | 0.0279 |
| Presentation in the rainy season (May – October) | 1.06 | 0.82 – 1.37 | 0.6688 |

**Supplementary Table 2. Results of a multivariable logistic regression model with WHO primary endpoint pneumonia on chest x-ray as the dependent variable**

The model includes data from 1,898 clinical pneumonia episodes

*PCV13 vaccinated defined as at least two doses of PCV13 (children aged between 0 – 11 months) or at least one dose of PCV13 (children aged ≥12 months).

†Comorbidities assessed: HIV infection, tuberculosis (currently / previously treated), asthma, heart disease, splenectomy.

| **Serotype** | **Carriage** | **Pneumonia** | **Total** | **OR** | **2.5% CI** | **97.5% CI** | **OR (rel.6B)*** | **2.5% CI (rel.6B)** | **97.5% CI (rel.6B)** |
| --- | --- | --- | --- | --- | --- | --- | --- | --- | --- |
| 6B | 144 | 151 | 295 | 1.090 | 0.827 | 1.433 | 1.000 | 1.000 | 1.000 |
| 6A | 131 | 105 | 236 | 0.970 | 0.709 | 1.321 | 0.890 | 0.858 | 0.922 |
| 19F | 103 | 96 | 199 | 0.984 | 0.705 | 1.369 | 0.903 | 0.852 | 0.955 |
| 15B/C | 110 | 66 | 176 | 0.685 | 0.478 | 0.972 | 0.628 | 0.577 | 0.678 |
| NT | 74 | 92 | 166 | 1.425 | 1.001 | 2.028 | 1.308 | 1.210 | 1.415 |
| 19A | 79 | 80 | 159 | 1.064 | 0.730 | 1.543 | 0.976 | 0.882 | 1.077 |
| 23F | 96 | 60 | 156 | 0.655 | 0.447 | 0.950 | 0.602 | 0.541 | 0.663 |
| 34 | 58 | 45 | 103 | 0.712 | 0.451 | 1.112 | 0.654 | 0.546 | 0.776 |
| 23A | 66 | 35 | 101 | 0.642 | 0.400 | 1.011 | 0.590 | 0.484 | 0.706 |
| 11A | 50 | 36 | 86 | 0.519 | 0.303 | 0.870 | 0.476 | 0.366 | 0.607 |
| 15A | 38 | 48 | 86 | 1.110 | 0.684 | 1.798 | 1.018 | 0.827 | 1.255 |
| 14 | 40 | 37 | 77 | 1.444 | 0.873 | 2.372 | 1.325 | 1.056 | 1.655 |
| 35C | 24 | 25 | 49 | 0.926 | 0.473 | 1.782 | 0.850 | 0.572 | 1.243 |
| 13 | 24 | 20 | 44 | 0.650 | 0.317 | 1.300 | 0.597 | 0.383 | 0.907 |
| 35B | 19 | 20 | 39 | 0.968 | 0.474 | 1.970 | 0.888 | 0.573 | 1.374 |
| 6C | 19 | 10 | 29 | 0.448 | 0.168 | 1.087 | 0.411 | 0.203 | 0.758 |
| 18C | 14 | 13 | 27 | 1.054 | 0.433 | 2.499 | 0.967 | 0.524 | 1.744 |
| 10A | 17 | 5 | 22 | 0.321 | 0.083 | 0.947 | 0.294 | 0.101 | 0.660 |
| 24F | 14 | 5 | 19 | 0.466 | 0.135 | 1.319 | 0.428 | 0.164 | 0.920 |
| 3 | 12 | 5 | 17 | 0.324 | 0.071 | 1.126 | 0.297 | 0.086 | 0.785 |
| 19B | 11 | 6 | 17 | 0.435 | 0.107 | 1.428 | 0.399 | 0.129 | 0.997 |
| 16F | 15 | 1 | 16 | 0.057 | 0.003 | 0.353 | 0.053 | 0.003 | 0.246 |
| 9V | 6 | 3 | 9 | 0.453 | 0.061 | 2.343 | 0.416 | 0.074 | 1.635 |
| *6D* | *7* | *0* | *7* | *0.000* | *-* | *41.733* | *0.000* | *-* | *29.120* |
| 7B | 4 | 3 | 7 | 0.594 | 0.057 | 3.933 | 0.545 | 0.068 | 2.744 |
| 10B | 2 | 5 | 7 | 4.253 | 0.867 | 30.784 | 3.903 | 1.049 | 21.480 |
| 35F | 3 | 4 | 7 | 0.849 | 0.105 | 5.862 | 0.779 | 0.127 | 4.090 |
| 38 | 3 | 4 | 7 | 1.522 | 0.280 | 8.535 | 1.397 | 0.338 | 5.955 |
| 9A | 3 | 2 | 5 | 0.237 | 0.020 | 2.238 | 0.218 | 0.025 | 1.561 |
| 17F | 3 | 2 | 5 | 1.028 | 0.131 | 6.482 | 0.943 | 0.158 | 4.523 |
| 18F | 3 | 2 | 5 | 0.806 | 0.069 | 6.403 | 0.739 | 0.083 | 4.468 |
| *33B* | *5* | *0* | *5* | *0.000* | *-* | *22642* | *0.000* | *-* | *15799* |
| 4 | 2 | 1 | 3 | 0.372 | 0.013 | 5.603 | 0.342 | 0.016 | 3.910 |
| 22F | 2 | 1 | 3 | 0.258 | 0.007 | 4.862 | 0.237 | 0.009 | 3.393 |
| 23B | 2 | 1 | 3 | 0.403 | 0.019 | 4.217 | 0.370 | 0.023 | 2.942 |
| 31 | 2 | 1 | 3 | 0.212 | 0.007 | 3.806 | 0.194 | 0.008 | 2.655 |
| *7C* | *2* | *0* | *2* | *0.000* | *-* | *7183023112* | *0.000* | *-* | *5012100306* |
| 18A | 1 | 1 | 2 | 0.752 | 0.018 | 30.532 | 0.690 | 0.022 | 21.304 |
| *18B* | *2* | *0* | *2* | *0.000* | *-* | *12757073627* | *0.000* | *-* | *8901507296* |
| *21* | *0* | *2* | *2* | *163841* | *0.000* | *-* | *150367* | *0.000* | *-* |
| *28F* | *2* | *0* | *2* | *0.000* | *-* | *12170426709* | *0.000* | *-* | *8492162490* |
| 33D | 1 | 1 | 2 | 1.632 | 0.058 | 46.198 | 1.498 | 0.070 | 32.235 |
| *35A* | *2* | *0* | *2* | *0.000* | *-* | *7183023112* | *0.000* | *-* | *5012100307* |
| *11D* | *1* | *0* | *1* | *0.000* | *-* | *2076756197573* | *0.000* | *-* | *1449098828210* |
| *15F* | *0* | *1* | *1* | *85292* | *0.000* | *-* | *78277* | *0.000* | *-* |
| *24A* | *1* | *0* | *1* | *0.000* | *-* | *8486537145081* | *0.000* | *-* | *5921653705365* |
| *33A* | *1* | *0* | *1* | *0.000* | *-* | *2076756197586* | *0.000* | *-* | *1449098828219* |
| *37* | *0* | *1* | *1* | *10666* | *0.000* | *-* | *9789* | *0.000* | *-* |

**Supplementary Table 3. Pneumococcal serotype detection from nasopharyngeal swab, by cohort (pneumonia = 2,209 swabs; carriage = 1,800 swabs)**

Odds Ratios (OR) and associated 95% confidence intervals (CI) are presented both raw and relative to serotype 6B, following adjustment for PCV13 immunisation status, recent antibiotic exposure, and age group. Values >100 have been rounded down to nearest whole number. Serotypes in red were associated with pneumonia (OR >1 with 95% CI >1, text in italics indicate detection only in this cohort yielding unreliable 95% CI), serotypes in green were associated with the out-patient carriage cohort (OR <1 with 95% CI <1, italics indicate detection only in this cohort yielding unreliable 95% CI).

*rel.6B: raw OR and 95% CI result divided by the values for serotype 6B, which was equally common in both cohorts.
